# Symptom- and Laboratory-Based Ebola Risk Scores to Differentiate Likely Ebola Infections

**DOI:** 10.3201/eid2311.170171

**Published:** 2017-11

**Authors:** Shefali Oza, Alieu A. Sesay, Neal J. Russell, Kevin Wing, Sabah Boufkhed, Lahai Vandi, Sahr C. Sebba, Rachael Cummings, Francesco Checchi

**Affiliations:** Save the Children International, Kerry Town, Sierra Leone (S. Oza, A.A. Sesay, N.J. Russell, K. Wing, S. Boufkhed, L. Vandi, S.C. Sebba, R. Cummings, F. Checchi);; London School of Hygiene and Tropical Medicine, London, United Kingdom (S. Oza, N.J. Russell, K. Wing, S. Boufkhed, F. Checchi);; Save the Children International, London (R. Cummings, F. Checchi)

**Keywords:** hemorrhagic fever, Ebola, disease outbreaks, risk, Sierra Leone, viruses, Ebola virus, Ebola virus disease, EVD

## Abstract

Rapidly identifying likely Ebola patients is difficult because of a broad case definition, overlap of symptoms with common illnesses, and lack of rapid diagnostics. However, rapid identification is critical for care and containment of contagion. We analyzed retrospective data from 252 Ebola-positive and 172 Ebola-negative patients at a Sierra Leone Ebola treatment center to develop easy-to-use risk scores, based on symptoms and laboratory tests (if available), to stratify triaged patients by their likelihood of having Ebola infection. Headache, diarrhea, difficulty breathing, nausea/vomiting, loss of appetite, and conjunctivitis comprised the symptom-based score. The laboratory-based score also included creatinine, creatine kinase, alanine aminotransferase, and total bilirubin. This risk score correctly identified 92% of Ebola-positive patients as high risk for infection; both scores correctly classified >70% of Ebola-negative patients as low or medium risk. Clinicians can use these risk scores to gauge the likelihood of triaged patients having Ebola while awaiting laboratory confirmation.

The 2014–2016 West Africa Ebola virus epidemic, unparalleled in spread for this disease, quickly overwhelmed the health systems of the 3 most affected countries (*1*). Ebola virus disease (EVD) can be difficult to initially identify even in a well-functioning health system because its early symptoms can closely mimic those of other common illnesses, such as malaria, typhoid, viral illness, and gastroenteritis (*2**,**3*). Thus, in an already weakened health system, the task of quickly but correctly identifying and isolating Ebola patients before laboratory test results are available is particularly challenging. This fact can result in missed opportunities to isolate infectious patients (through incomplete screening sensitivity) and expose non-Ebola patients to nosocomial infection (through incomplete specificity).

Currently, the most common laboratory test to identify EVD relies on a reverse transcription PCR (*2*), which is not a rapid point-of-care (POC) test but instead requires substantial laboratory infrastructure. In the West Africa outbreak, patient blood samples were typically sent to off-site laboratories set up through the international response. Although the test itself can be done in hours, the round trip from a health facility to the laboratory often took >3 days, especially during the peak of the epidemic (*4*). Although a few rapid POC EVD diagnostics were developed and field-tested during this outbreak, they are not yet ready for widespread commercial use (*5**,**6*).

In lieu of rapid POC EVD tests to identify EVD-positive cases, a standardized EVD case definition from the World Health Organization (WHO) was used during the epidemic as the primary tool for initially identifying potential EVD patients (*7*). Because false negatives for EVD put patients and their communities at great risk, this case definition is broad (high sensitivity/low specificity) (*8*). A broad case definition is also useful for epidemic surveillance. Patients meeting the case definition, which is based on symptoms and potential exposure, were sent to holding centers for EVD testing and isolation. However, the broad case definition meant that negative and positive EVD patients were mixed together, often for days, until their test results were available and treatment facilities had beds for the positive patients. Although some holding centers tried to separate suspect patients based on wet (i.e., diarrhea or vomiting) versus dry symptoms, this crude separation can expose Ebola-negative patients, particularly those with wet symptoms, to a higher risk of nosocomial infection.

Thus, an Ebola risk score that rapidly further differentiates the likelihood of Ebola infection beyond the case definition could be beneficial. Risk scores based on noninvasive information, such as demographic characteristics and symptoms, are practical tools for diagnostic prediction models because the predictors are comparatively simple to ascertain. Such risk scores are especially useful in resource-limited settings because a range of persons, from community health workers to clinicians, can quickly obtain the necessary patient information (*9**,**10*). Symptom-based risk scores have been used to identify patients at higher risk of various diseases (e.g., pulmonary tuberculosis, gastric cancer) or outcomes (e.g., predicting mortality in sick children) in resource-limited settings (*11**–**13*). However, symptom-based risk scores are limited by the accuracy of symptom reporting and the predictive power of those symptoms (*14**,**15*). Additional variables, such as laboratory tests, can improve the accuracy of risk scores, at the cost of less versatility (*16**,**17*). One solution has been to develop risk scores with optional additional variables to improve accuracy when those variables can be ascertained (*13*). As rapid POC laboratory devices such as the Piccolo Xpress (Abaxis, Inc., Union City, CA, USA) and i-STAT (Abbott Point of Care, Princeton, NJ, USA) analyzers have become more common even in low-income settings (*18*), adding optional laboratory tests to risk scores has become feasible.

We analyzed data from the Kerry Town Ebola treatment center (ETC) in Sierra Leone to develop 2 Ebola risk scores. The first uses symptom data and the second incorporates biochemistry laboratory tests to improve prediction accuracy. The goal of these risk scores is to supplement the broad WHO case definition by further separating triaged patients on the basis of their likelihood of being EVD positive.

## Methods

### Study Design and Patient Population

This research consisted of a retrospective cohort study of deidentified data on patients from November 5, 2014, through March 31, 2015, at the Kerry Town ETC in Sierra Leone. This 80-bed ETC, based in the Western Area Rural district, was operated by Save the Children International in partnership with the United Kingdom and Sierra Leone governments.

The patient population at the ETC consisted of patients with suspected or confirmed EVD, mostly from the nearby Western Area Urban and Western Area Rural districts. The ETC featured dry and wet wards for suspected Ebola patients (suspect wards) without a prior EVD test result and treatment wards for those confirmed to have EVD. Patients already confirmed to have EVD at previous holding centers were admitted directly to the confirmed wards. Suspected patients who met the admission criteria at triage were admitted to suspect wards while awaiting their EVD test results. All confirmed and suspected patients received on-site EVD tests, with results in <24 hours from admission. Suspected patients who tested positive were transferred to the confirmed wards; those testing negative were discharged or retested for up to 3 days before being discharged. All ETC patients with information recorded on basic demographic details and baseline symptoms were included in this study. The Sierra Leone Ethics and Scientific Review Committee and the London School of Hygiene and Tropical Medicine in the United Kingdom granted ethical approval for this study.

### Data Collection

We used only data routinely collected for patient care on standardized clinical record forms. Referred patients often arrived with forms from their previous holding centers and/or a standardized case investigation form containing demographic and epidemiologic characteristics, as well as symptoms at admission (*7*).

We transcribed data from the paper clinical records into electronic format using Excel version 14.5.2 (Microsoft, Redmond, WA, USA). We then imported these data into Stata version 12 (https://www.stata.com) for statistical analyses.

### Data Input and Cleaning

The outcome measure was EVD, confirmed by the on-site Public Health England laboratory using a reverse transcription PCR. For potential predictors of positive EVD test outcomes, we investigated 14 commonly recorded symptoms and analyzed these as binary variables. We recorded a symptom as present if it was checked as present on the case investigation, triage, or baseline admission forms. In an additional analysis, we included among the potential predictors 13 biochemistry laboratory tests performed by the on-site UK Ministry of Defense laboratory using a Piccolo Xpress device, which can yield rapid results (≈12 minutes) at the point of care. For the purpose of our analysis, we converted the test results into categorical variables based on low and high abnormal test ranges.

We performed multiple imputation by chained equations (*19*) for missing laboratory results based on the missing at random assumption. We used predictive mean matching with 20 iterations and included all the symptoms, the outcome variable, age, and sex as factors for the imputation.

### Model Building

We built our symptom-based predictive model as follows. First, we performed univariable logistic regressions of each symptom against EVD outcome. We retained any symptom that had a p value of <0.40 for further analysis. We chose this lenient cutoff to balance the poor performance of diagnostic prediction models when relying solely on p values (*20*) against the need to reduce the number of symptom combinations being investigated. We used 10-fold cross-validation to assess the best out-of-sample fit for all combinations of symptoms retained in the analysis after the univariable analysis. We chose to use cross validation because commonly used stepwise procedures for model selection have come under widespread criticism for several reasons, including overfitting, p value exaggeration, and biased coefficient estimates (*21**,**22*). Moreover, we were interested in selecting symptom combinations with better out-of-sample performance for use outside our patient dataset. 

We used standard methods for cross-validation (*23*). First, we randomly partitioned the dataset into 10 equal subsamples. We then ran multivariable logistic regressions of each symptom combination against EVD outcome on 90% of the data (i.e., the training dataset), and used the results to predict the probability of EVD for each observation in the remaining 10% of the dataset (i.e., the validation dataset). For each symptom combination, we created receiver operating characteristic (ROC) curves and calculated the area under the curve (AUC) for each of the 10 validation datasets. The AUC is a standard measure of accuracy of test performance, with 1 indicating a perfect model and 0.5 indicating no better than random guessing (*24*). We took the median of the 10 AUCs as the overall goodness of fit for each candidate symptom combination model. We chose the best-fit model based on which model had the highest out-of-sample median AUC.

### Development of Risk Score

We used previously established methods (*13**,**25*) to develop an easy-to-use Ebola symptom-based risk (ESR) score using the selected model. First, we ran a multivariable logistic regression of the model against EVD. We then assigned integer scores to individual symptoms based on their regression coefficients, with a score of 1 for coefficients <1 and a score of 2 for coefficients >1, while keeping the original sign. We calculated a patient’s overall ESR score by adding the integer scores for the symptoms present in that patient. We then mapped the number of patients by true EVD status at each integer level of the ESR score. We evaluated the score by calculating the sensitivity, specificity, positive predictive value (PPV), and negative predictive value (NPV) for each level by designating all patients below that level as EVD negative and those at or above it as EVD positive. Finally, we determined low-, medium-, and high-risk categories by identifying risk thresholds that first maximized the number of true EVD positives in the high-risk category and minimized them in the low-risk category, and then best limited the true EVD negatives in the high-risk category.

### Adding Laboratory Tests to the Ebola Risk Score

We performed additional analysis on patients with >1 non-Ebola laboratory result. We used univariable logistic regressions of the categorical laboratory test values against EVD outcome to retain laboratory tests with a p value of <0.40. For each possible combination of tests, we performed a multivariable logistic regression, this time adjusting for symptoms in the ESR score, and assigned an integer score to the laboratory tests using the same rule as that for the ESR score. We then used the net reclassification improvement (NRI) metric (*26*) and reclassification tables (*27*) to evaluate which laboratory test combinations best improved the symptom-based Ebola risk score. Of the 10 models with the highest NRI, we chose the model with the largest summed improvement in categorizing EVD-positive and EVD-negative patients within the high-risk category of the ESR score. We chose this metric to prioritize an improvement in sensitivity while not overly sacrificing specificity. We added the individual laboratory test integer scores to the ESR score for a final Ebola symptom- and laboratory-based (ESLR) score. To determine whether the ESLR score was an improvement over the ESR score, we visually compared their ROC curves and tested the statistical significance of the difference in AUCs (*28*).

### Internal Validation

We performed internal validation of our risk scores using the bootstrap method to correct for overoptimism (*29*). We drew 1,000 bootstrap samples with replacement and calculated the AUC for the best model in each sample and the corresponding AUC in the full dataset. We then took the difference of these mean AUCs and corrected for overoptimism by subtracting this total from the AUC of our risk score. We performed this process separately for the ESR and ESLR scores.

## Results

Of the 456 patients who were admitted to the Kerry Town ETC with suspected or confirmed EVD, we excluded 32 patients (7.0%): 31 had no baseline forms or minimal/no symptoms recorded, and 1 died before an EVD test could be conducted. Of the remaining 424 patients, samples from 252 tested positive for EVD and samples from 172 tested negative. Basic demographic characteristics and outcomes of the patients are provided in Table 1, and frequencies of clinical symptoms and the univariable logistic regression results by EVD status are shown in Table 2. The 10 symptoms with univariable p values <0.40 gave rise to 1,024 candidate symptom combinations for final model selection.

**Table 1:** Basic characteristics and outcomes of patients by EVD status at the Kerry Town ETC, Sierra Leone, 2014–2015*

Characteristic	EVD negative, n = 172	EVD positive, n = 252
Sex, no. (%)		
F	69 (40.1)	144 (57.1)
M	103 (59.9)	108 (42.9)
Median age, y (IQR)	27 (20–40)	25 (14–35)
Mode of arrival to facility, no. (%)		
Ambulance (referral)	95 (55.2)	242 (96.0)
Walk-in	77 (44.8)	10 (4.0)
Median days between onset of symptoms and admission (IQR)	3 (2–6)	3 (2–5)
Median length of stay at ETC, d (IQR)	1 (1–2)	6 (3–11)
Deaths, no. (case fatality ratio, %)	12 (7.0)	107 (42.5)

*ETC, Ebola treatment center; EVD, Ebola virus disease; IQR, interquartile range.

**Table 2:** Patient clinical symptoms by Ebola status at the Kerry Town ETC, Sierra Leone, 2014–2015*

Symptom	EVD negative, no. (%), n = 172	EVD positive, no. (%), n = 252	Univariable logistic regression	
Coefficient (95% CI)	p value
Fever†	165 (95.9)	230 (91.3)	−0.81 (−1.69 to 0.06)	0.068
Headache†	132 (76.7)	161 (63.9)	−0.62 (−1.06 to −0.19)	0.005
Fatigue	158 (91.9)	226 (89.7)	−0.26 (−0.94 to 0.42)	0.452
Joint/muscle pain†	146 (84.9)	182 (72.2)	−0.77 (−1.27 to −0.27)	0.003
Diarrhea†	68 (39.5)	165 (65.5)	1.06 (0.66 to 1.47)	<0.001
Bleeding	20 (11.6)	27 (10.7)	−0.09 (−0.71 to 0.52)	0.769
Difficulty breathing†	93 (54.1)	48 (19.1)	−1.61 (−2.04 to −1.18)	<0.001
Nausea/vomiting†	95 (55.2)	173 (68.7)	0.57 (0.17 to 0.98)	0.005
Abdominal pain	111 (64.5)	162 (64.3)	−0.01 (−0.42 to 0.39)	0.958
Hiccups†	39 (22.7)	42 (16.7)	−0.38 (−0.87 to 0.10)	0.124
Swallowing pain	56 (32.6)	90 (35.7)	0.14 (−0.27 to 0.55)	0.502
Loss of appetite/anorexia†	156 (90.7)	174 (69.1)	−1.47 (−2.05 to −0.90)	<0.001
Conjunctivitis†	44 (25.6)	122 (48.4)	1.00 (0.58 to 1.43)	<0.001
Rash†	6 (3.5)	16 (6.4)	0.63 (−0.33 to 1.59)	0.199

*ETC, Ebola treatment center EVD; Ebola virus disease. †p<0.40 and thus retained to construct candidate symptom combinations.

Using the cross-validation model selection, we obtained the best out-of-sample fit with a 6-symptom model comprising headache, diarrhea, difficulty breathing, nausea/vomiting, loss of appetite, and conjunctivitis. The median out-of-sample AUC for this model was 0.84 (interquartile range [IQR] 0.79–0.86). The validation analysis yielded a correction of 0.012, resulting in an internally validated AUC of 0.83 for the ESR score, which is considered excellent discrimination (*30*).

We found >1 non-EVD laboratory result for 309 patients. Of these, the percentage of missing laboratory results ranged from 5.1% for glucose to 15.2% for total bilirubin and aspartate aminotransferase. Visual inspection of the observed and imputed data suggested that the imputation worked well (Technical Appendix Figures 1–13). We excluded glucose and albumin because of p values >0.40 and thus retained 11 laboratory tests for consideration (Technical Appendix Table 1).

According to the NRI and reclassification tables (Technical Appendix Table 2), the best combination of additional laboratory tests was creatinine, creatine kinase (CK), alanine aminotransferase (ALT), and total bilirubin. The median out-of-sample AUC for the ESLR score was 0.91 (IQR 0.89–0.92). The internally validated AUC for the ESLR score was 0.90, which is considered outstanding discrimination (*30*).

**Figure 1:**
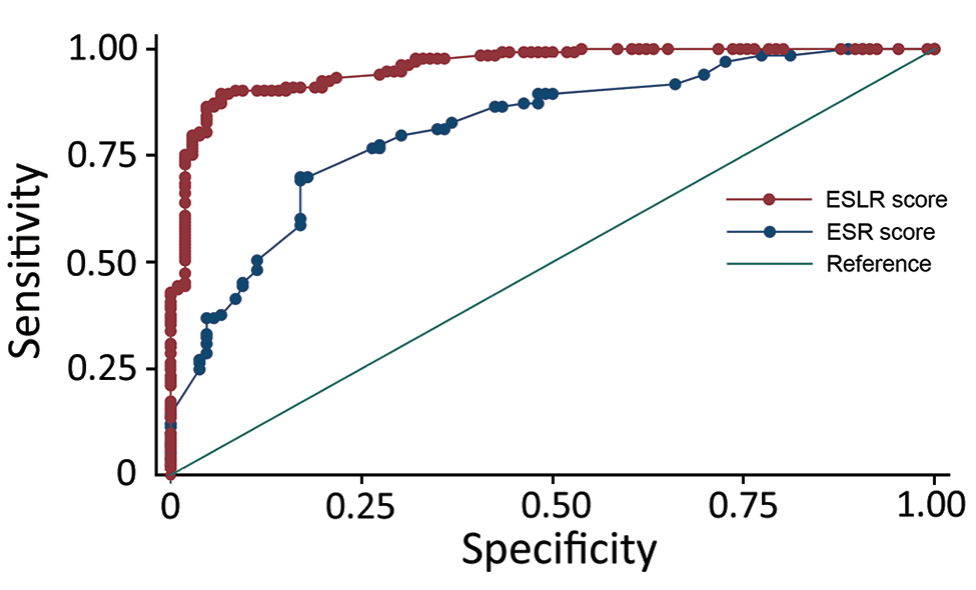
Receiver operating characteristic curves for ESR and ESLR scores to determine risk for infection in suspected Ebola patients. ESLR, Ebola symptom- and laboratory-based risk; ESR, Ebola symptom-based risk.

Table 3 shows the multivariable model results, as well as the assigned integer scores for individual symptom and laboratory tests. An individual patient’s ESR score could range from –3 to +5 and the ESLR score from –4 to +9. The trade-off between sensitivity and specificity for each score level was generally better for the ESLR than for the ESR score (Table 4). The difference in the AUC of the ROC indicated that the ESLR score was a significant improvement (p<0.001) over the ESR score (Figure 1).

**Table 3:** Factors included in ESR and ESLR scores to determine risk for infection in suspected Ebola patients*

Factor	Coefficient (95% CI) from multivariable model†	p value	Score value
Symptoms, for ESR and ESLR scores			
Conjunctivitis	1.44 (0.93 to 1.95)	<0.001	+2
Diarrhea	1.11 (0.60 to 1.61)	<0.001	+2
Nausea/vomiting	0.78 (0.24 to 1.31)	0.005	+1
Headache	−0.45 (−0.98 to 0.09)	0.103	−1
Difficulty breathing	−1.60 (−2.11 to 1.10)	<0.001	−1
Loss of appetite	−1.90 (−2.60 to −1.20)	<0.001	−1
Laboratory tests if available, for ESLR score only			
Alanine transaminase >48 U/L	3.83 (2.67 to 5.00)	<0.001	+2
Creatine kinase >380 U/L	1.78 (0.73 to 2.84)	0.001	+2
Creatinine >106 μmol/L	−1.15 (−2.21 to −0.09)	0.033	−1
Total bilirubin >27 μmol/L	−1.81 (−3.24 to −0.39)	0.012	−1

*ESLR, Ebola symptom- and laboratory-based risk; ESR, Ebola symptom-based risk.  †Laboratory tests have been adjusted for the symptom predictors. Coefficient values are before normalization.

**Table 4:** Sensitivity, specificity, positive predictive value, and negative predictive value of ESR and ESLR scores to determine risk for infection in suspected Ebola patients*

Score	% EVD negative	% EVD positive	Sensitivity† (95% CI)	Specificity† (95% CI)	PPV† (95% CI)	NPV† (95% CI)
ESR score, range −3 to +5						
−3	8	0	100 (100–100)	0 (0–0)	59 (55–64)	NA
−2	16	4	100 (100–100)	8 (4–12)	61 (57–66)	100 (100–100)
−1	19	10	96 (94–98)	24 (18–31)	65 (60–70)	81 (70–91)
0	33	14	86 (81–90)	43 (36–50)	69 (64–74)	67 (59–76)
1	13	19	72 (66–77)	76 (70–83)	82 (76–87)	65 (58–71)
2	6	24	53 (47–59)	89 (84–94)	88 (82–93)	56 (50–62)
3	5	21	29 (23–35)	95 (92–98)	90 (84–97)	48 (43–53)
4	0	7	8 (5–11)	100 (100–100)	100 (100–100)	43 (38–47)
5	0	1	1 (0–3)	100 (100–100)	100 (100–100)	41 (36–46)
ESLR score, range −4 to +9						
−4	1	0	100 (100–100)	0 (0–0)	56 (50–62)	NA
−3	10	1	100 (100–100)	1 (0–2)	56 (51–62)	100 (100–100)
−2	16	1	99 (98–100)	10 (5–15)	59 (53–64)	93 (81–100)
−1	19	3	99 (97–100)	26 (19–34)	63 (57–69)	95 (88–100)
0	25	3	95 (92–99)	46 (37–54)	69 (63–75)	89 (81–96)
1	11	8	92 (88–96)	71 (63–78)	80 (74–85)	87 (81–94)
2	7	14	84 (79–90)	82 (75–88)	85 (80–91)	80 (74–87)
3	9	14	70 (63–77)	89 (84–94)	89 (84–94)	70 (63–77)
4	1	20	55 (48–63)	98 (95–100)	97 (94–100)	63 (57–70)
5	1	17	36 (29–43)	99 (97–100)	97 (93–100)	55 (48–61)
6	0	10	18 (13–24)	100 (100–100)	100 (100–100)	49 (43–55)
7	0	7	9 (4–13)	100 (100–100)	100 (100–100)	46 (41–52)
8	0	1	2 (0–4)	100 (100–100)	100 (100–100)	44 (39–50)
9	0	1	1 (0–3)	100 (100–100)	100 (100–100)	44 (39–50)

*ESLR, Ebola symptom- and laboratory-based risk; ESR, Ebola symptom-based risk; EVD,  Ebola virus disease; NPV, negative predictive value; PPV,  positive predictive value.  †Based on greater or equal to the score (e.g., sensitivity for a score of 0 means that those with a score of 0 or higher were considered to be EVD positive).

**Figure 2:**
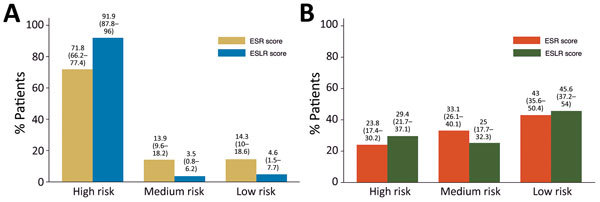
Suspected Ebola patients categorized as high-, medium-, and low-risk by ESR and ESLR scores, Kerry Town Ebola treatment center, Sierra Leone, 2014–2015. A) EVD-positive patients; B) EVD-negative patients. ESLR, Ebola symptom- and laboratory-based risk; ESR, Ebola symptom-based risk. Numbers in parentheses indicate 95% CIs.

We classified the ESR and ESLR scores as low risk if negative, medium risk if 0, and high risk if positive. Using the ESR score, we categorized 71.8% (95% CI 66.2%–77.4%) of EVD-positive patients as high risk and 14.3% (95% CI 10.0%–18.6%) as low risk (Figure 2, panel A). The ESLR score was more discriminant, with 91.9% (95% CI 87.8%–96.0%) of EVD positive patients considered high risk and 4.6% (95% CI 1.5%–7.7%) low risk using this score. Similar percentages of EVD-negative patients were categorized as low risk with the ESR (43.0%, 95% CI 35.6%–50.4%) versus ESLR (45.6%, 95% CI 37.2%–54.0%) scores (Figure 2, panel B). The ESR score performed better among EVD-negative patients classified as high risk, but not significantly so, with 23.8% (95% CI 17.4%–30.2%) for the ESR score compared with 29.4% (95% CI 21.7%–37.1%) for the ESLR score.

## Discussion

We developed Ebola risk scores, based on reported symptoms and, optionally, complementary POC laboratory tests, to help categorize suspected Ebola patients into low-, medium-, and high-risk categories. These risk scores can be used after applying the case definition to further separate suspected Ebola patients at triage based on their likelihood of having EVD.

The ESR and ESLR scores generally performed well. Of suspected patients whose specimens tested positive, >70% were categorized as high risk by the ESR score and >90% as high risk by the ESLR score. This result means that most true -positive patients could have been correctly separated from those who ultimately tested negative. About one quarter of EVD-negative patients were classified as high risk, but this trade-off was necessary to ensure that most true positives were correctly deemed high risk. Unfortunately, we do not have true diagnoses available for these non-Ebola patients to determine whether some illnesses were more associated with a high-risk classification than others. Compared with our results, we found low specificity (4.7%, 95% CI 1.5%–7.8%) and NPV (18.2%, 95% CI 6.8%–29.6%) when applying the symptom component of the WHO case definition (inexplicable bleeding or fever plus 3 of 10 symptoms) (*31*) to our patients. Both the ESR and ESLR scores can substantially improve upon these criteria. For example, using the threshold of >2 for the ESLR score resulted in 81.6% (95% CI 75.1%–88.1%) specificity and 80.4% (95% CI 73.8%–87.0%) NPV.

Although laboratory test results are generally of higher quality than self-reported symptoms, we included them only as optional additions to our risk score, which permits better accuracy while retaining the versatility of using only the symptom-based score in places without necessary laboratory equipment. The improvement with laboratory tests can be substantial; in our study, 21.3% of EVD-positive patients were newly classified as high risk when using the ESLR score instead of the ESR score. With the rise of POC tests in low-resource settings, risk scores that rely on laboratory tests are becoming more feasible. For example, we included only laboratory tests that are available using the rapid POC Piccolo Xpress device, which was used at our site and by others during the West Africa Ebola outbreak (*32**,**33*).

Although risk scores have become a common tool for stratifying and predicting risk, we found only one previous study that developed a risk score for Ebola (*34*). That study, based on data from a Liberian ETC in 2014, was a notable first step for Ebola risk scores, but it did not include laboratory tests and had a smaller sample size. This difference may explain why our ESR and ESLR scores appear to have better sensitivity/specificity trade-offs.

We were constrained by the amount and quality of patient data because the data were collected during a challenging emergency response and only essential clinical, epidemiologic, and demographic information was collected for each patient. Thus, some data that may have improved our Ebola risk score, such as other symptoms or detailed exposure information, were unavailable. For example, we had to exclude exposure as a potential predictor because of questionable quality and large amounts of missing data for this variable.

Our study is based on a patient population at 1 treatment center; these patients may not be representative of the overall population of suspected Ebola cases in West Africa or in future Ebola outbreaks. Additionally, the distribution of patient characteristics may be different at a general medical facility or outside of an epidemic. The small correction factor from the internal validation exercise suggests that our scores would work similarly in a different epidemic study population. Ideally, our Ebola risk score should be externally validated against data from a future outbreak or, if made available, from this one. Although other common Ebola strains have similar reported symptoms to this Zaire strain (*35*), our scores should not be used for them without testing/validation. In general, the techniques presented here could be used to develop new risk scores for such strains or other hemorrhagic fevers. Given the good internal validation and the rare inclusion of high-quality POC laboratory tests, however, we believe that our work is a step toward having an accurate Ebola risk score.

Our risk scores cannot replace the WHO case definition or actual diagnostic testing. They can, however, help fill the gap between a broad case definition and an often-lengthy diagnostic process, which is valuable for several reasons. First, our risk scores can be used to more accurately separate likely negative from likely positive Ebola patients after initial screening with the WHO case definition. For example, patients could be physically separated into low-, medium-, and high-risk suspect wards based on their risk score while awaiting Ebola test results. The higher-risk wards could then have further protections, such as additional barriers between patients or separation of wet and dry patients. This distinction is beneficial to both Ebola-negative patients and their communities because it reduces the risk of nosocomial infection that could be spread back to the community. Second, Ebola is a rapidly progressing disease, with typically only 6 to 16 days between onset of symptoms and death (*2*). Therefore, more accurately identifying likely positive cases while awaiting test results can mean earlier focus on and treatment of true positives during a response with limited resources. Third, with temporary Ebola holding and treatment centers now closed in West Africa, new cases there and elsewhere are likely to be screened at a wide range of places within the existing health system, from the community to local health centers to regional hospitals. When Ebola is rare, we still expect large numbers of patients to meet the WHO case definition because of the broad symptom list associated with EVD. An Ebola risk score can thus be useful in giving more precise information about risk to community health workers and clinicians. Finally, an advantage of using a risk score like ours is that, as the epidemic evolves, cutoffs for patient triage and categorization can be modified in real time (e.g., using Table 4) to reflect a changing emphasis on sensitivity versus specificity.

Given the danger Ebola poses, classifying the risk of suspected Ebola patients is essential. Until a reliable rapid POC diagnostic for Ebola is readily available in low-resource settings, a flexible risk score that is easy to implement can be a useful tool for further triaging patients. Even though outbreaks of poorly understood but dangerous infectious diseases will continue in the future, developing such risk scores can help inform the difficult choices that healthcare workers must make during these emergencies.

Technical Appendix Additional details for the various analyses, including laboratory test imputation analyses, laboratory test results by EVD status, and reclassification table for ESR versus ESLR scores.
