# Supplementary material for: Symptom- and Laboratory-Based Ebola Risk Scores to Differentiate Likely Ebola Infections
Source: Emerg Infect Dis. 2017 Nov;23(11):1792–9. doi: 10.3201/eid2311.170171 (PMC5652431; doi:10.3201/eid2311.170171)
Supplement: Technical Appendix — Additional details for the various analyses, including laboratory test imputation analyses, laboratory test results by EVD status, and reclassification table for ESR versus ESLR scores. [file 17-0171-Techapp-s1.pdf]

Article DOI: https://doi.org/10.3201/eid2311.170171

Symptom- and Laboratory-Based Ebola Risk Scores to Differentiate Likely Ebola Infections

Technical Appendix

This Technical Appendix contains additional details for the various analyses in the main article. This document is divided into 3 sections:

1. Laboratory test imputation analyses

2. Patient laboratory test results by Ebola virus disease (EVD) status

3. Reclassification table for Ebola symptom-based risk (ESR) versus Ebola symptom- and laboratory-based risk (ESLR) scores

Laboratory Test Imputation Analyses

Technical Appendix Figures 1–13. Observed, imputed, and completed data for 20 imputation iterations of laboratory tests.

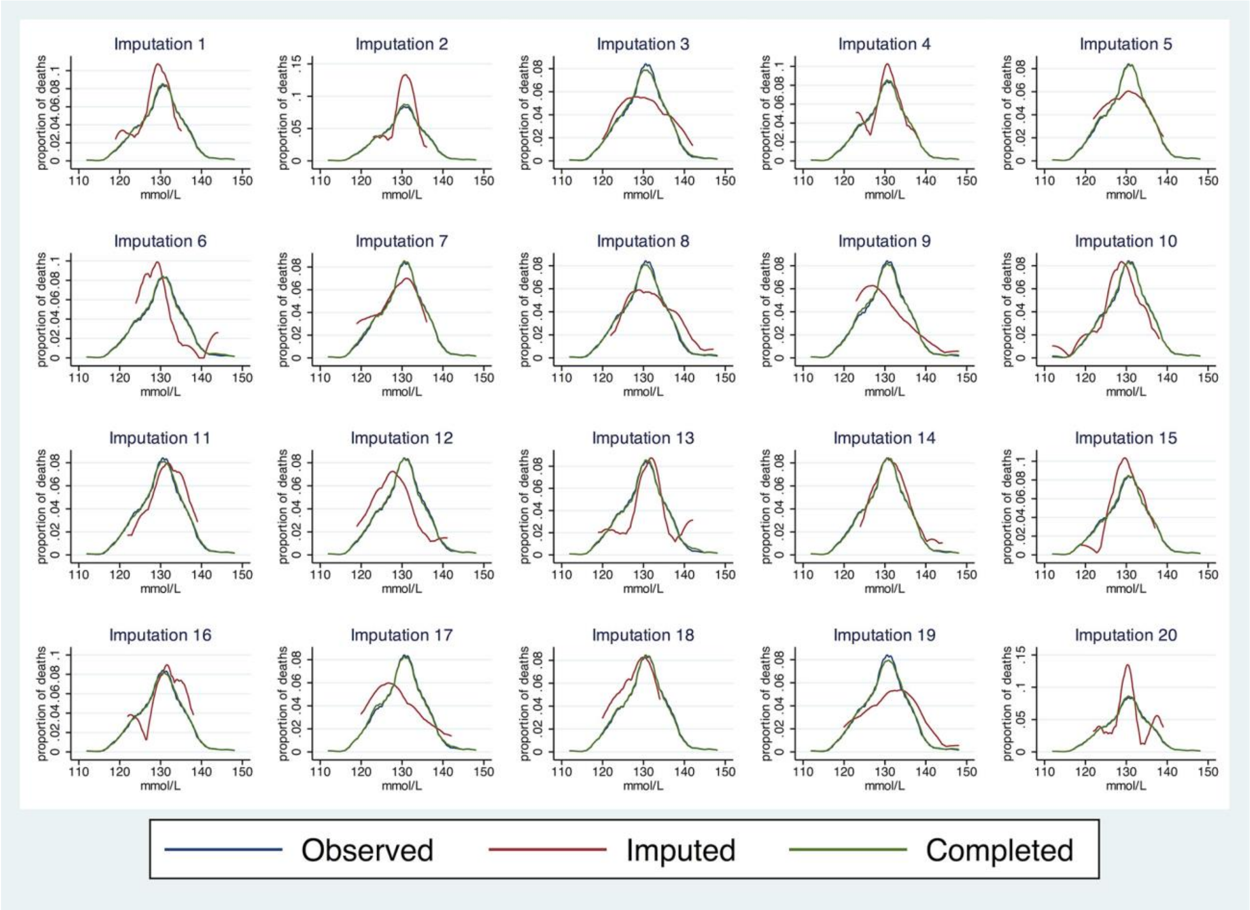

Technical Appendix Figure 1. Sodium: missing = 5.5% (17/309); Ebola+: 1/173 (0.6%); Ebola–: 16/136 (11.8%)

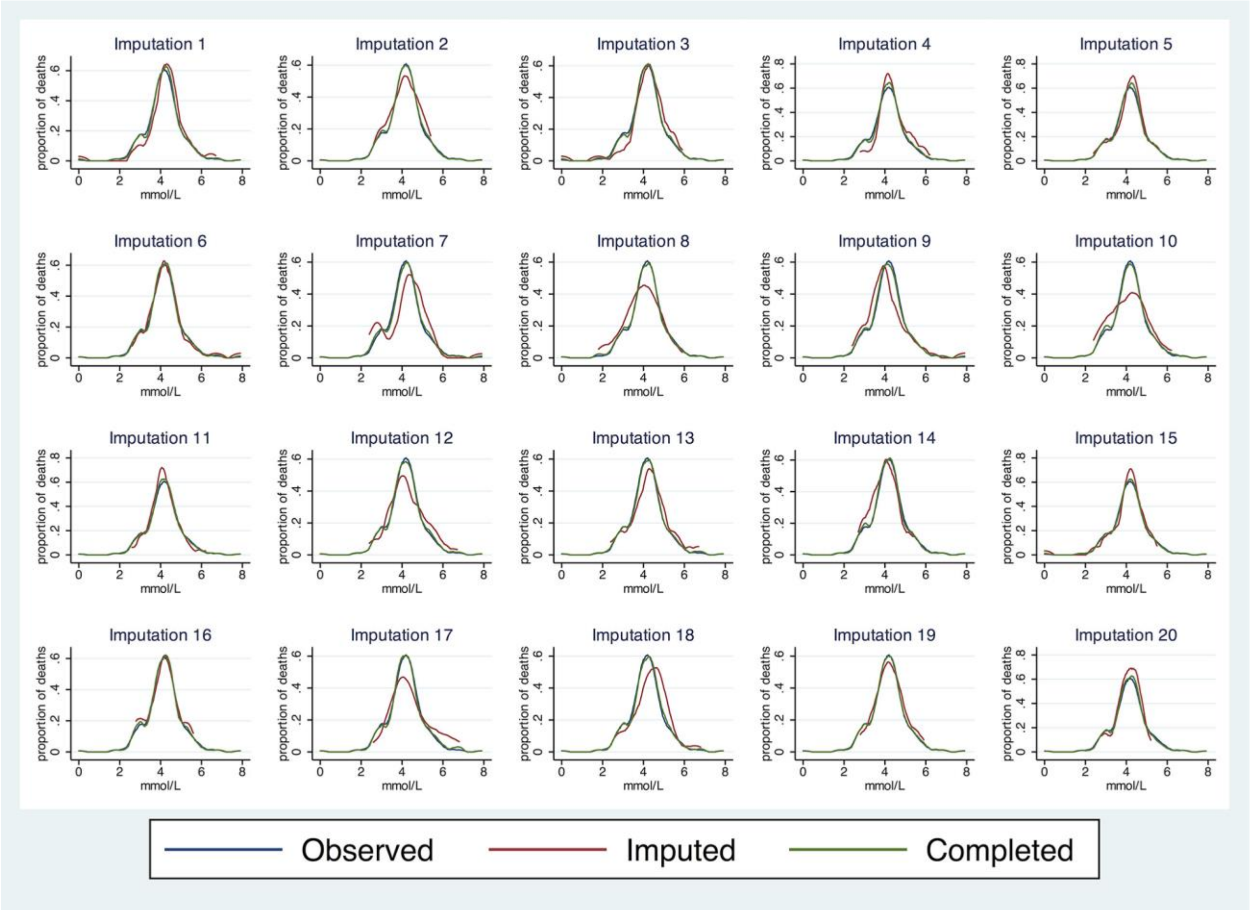

Technical Appendix Figure 2. Potassium: missing = 14.9% (46/309); Ebola+: 25/173 (14.5%); Ebola–: 21/136 (15.4%)

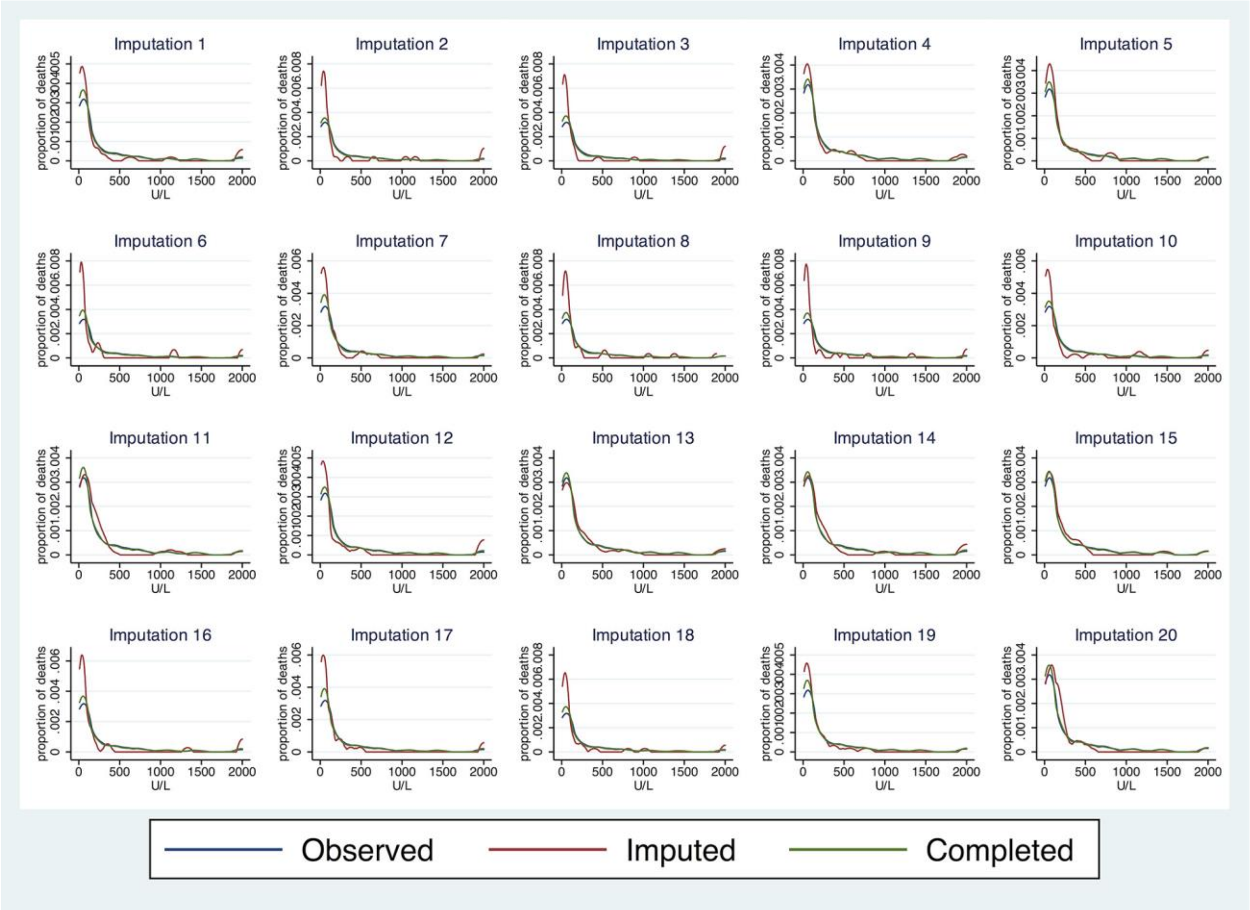

Technical Appendix Figure 3. Alanine aminotransferase (ALT): missing = 12.0% (37/309); Ebola+: 10/173 (5.8%); Ebola–: 27/136 (19.9%)

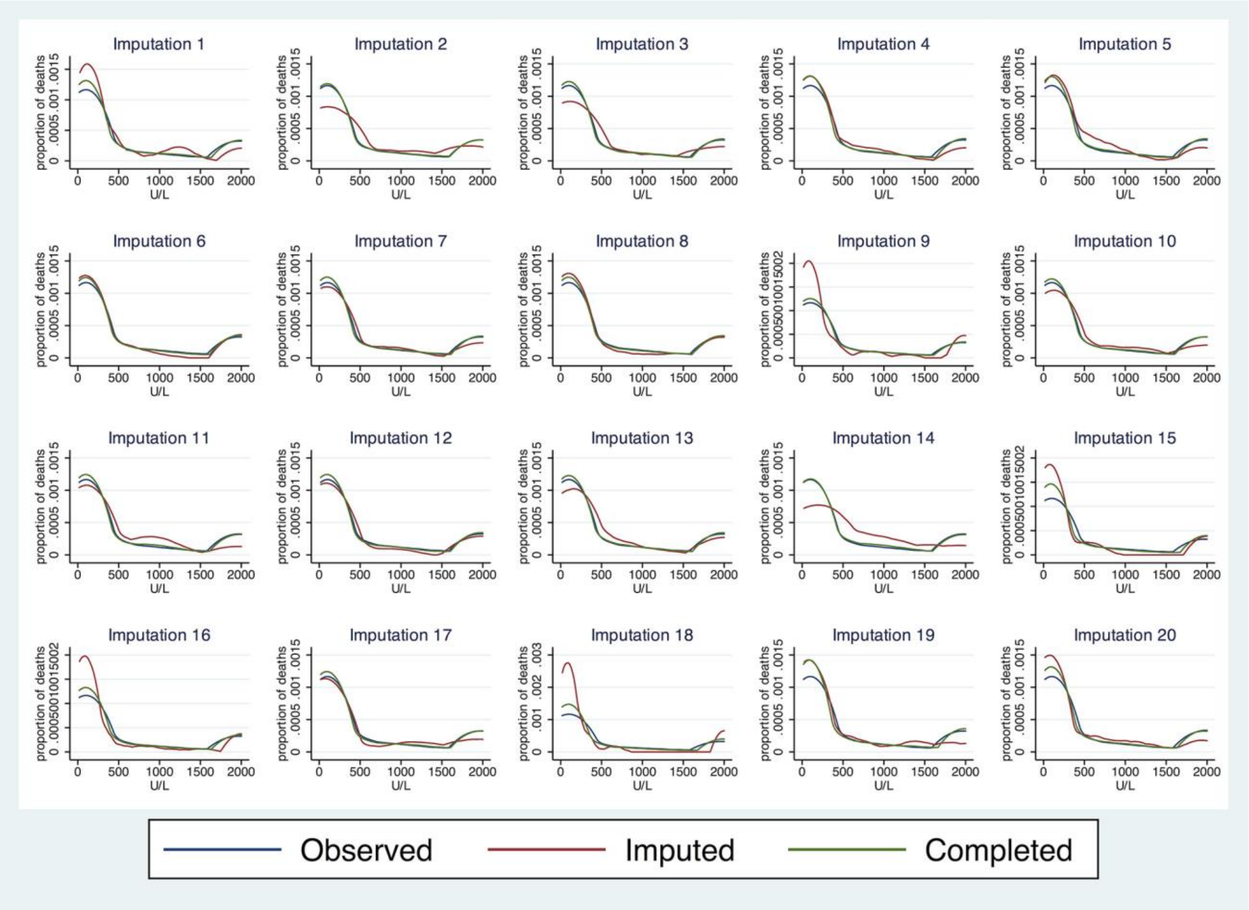

Technical Appendix Figure 4. Aspartate aminotransferase (AST): missing = 15.2% (47/309); Ebola+: 20/173 (11.6%); Ebola–: 27/136 (19.9%)

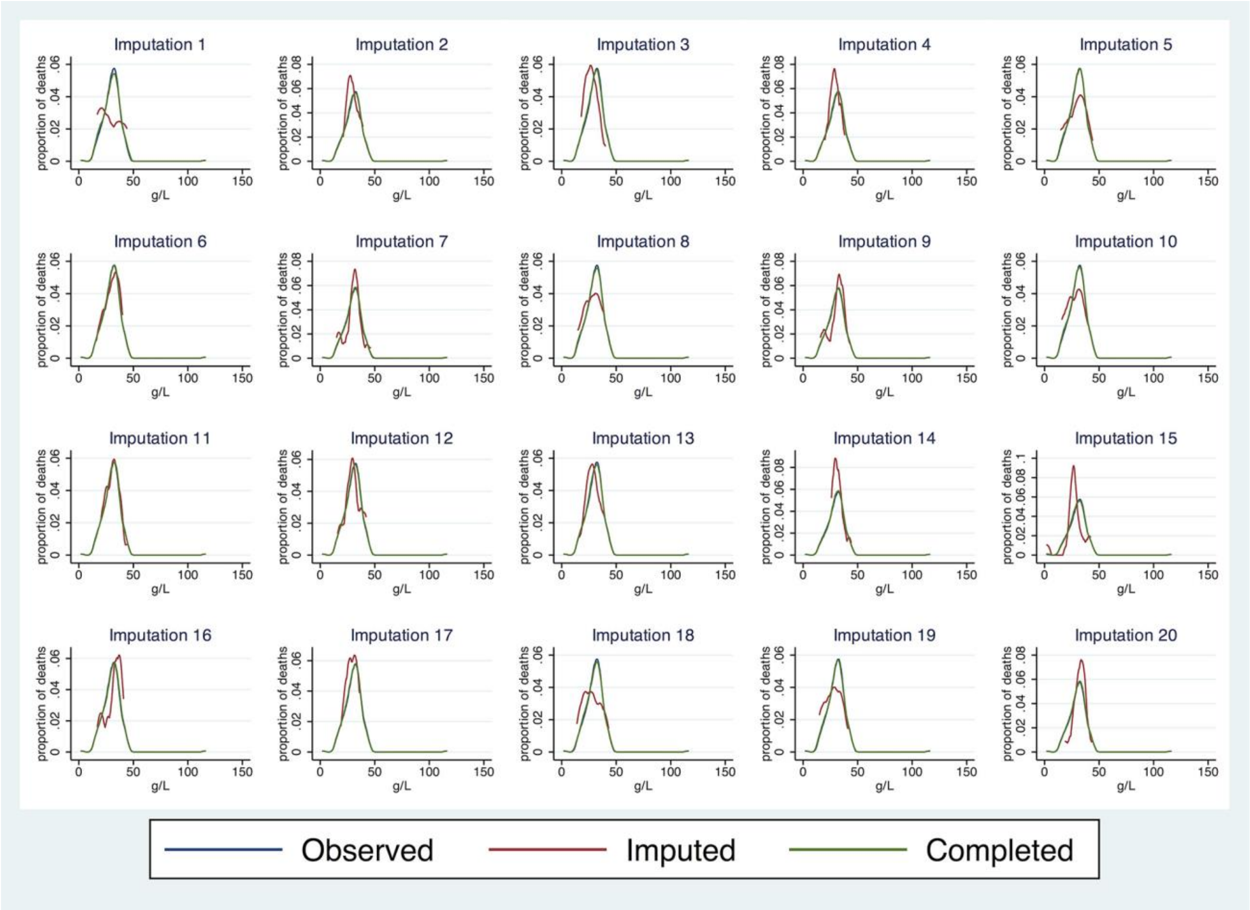

Technical Appendix Figure 5. Albumin: missing = 5.5% (17/309); Ebola+: 2/173 (1.2%); Ebola–: 15/136 (11.0%)

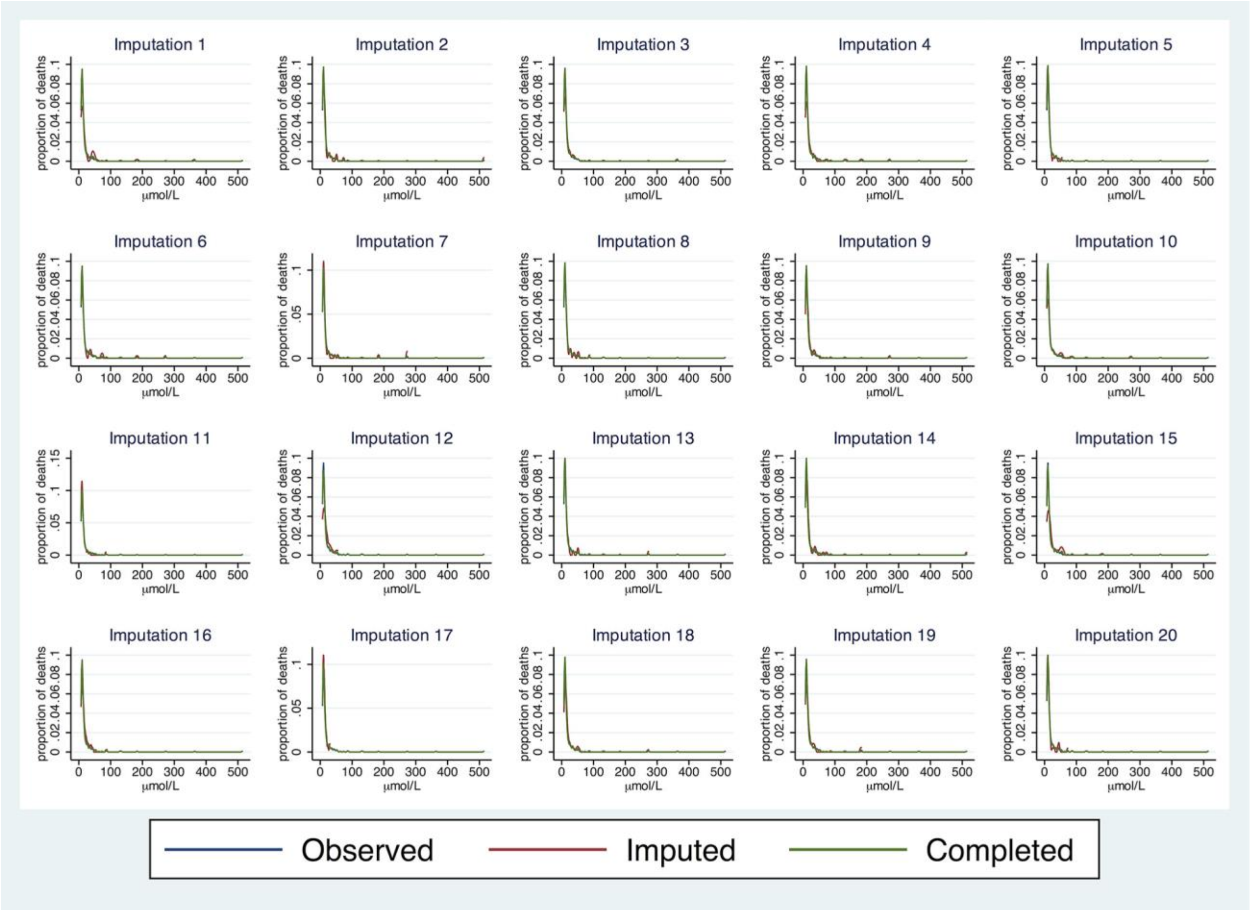

Technical Appendix Figure 6. Total bilirubin: missing = 15.2% (47/309); Ebola+: 22/173 (12.7%); Ebola–: 25/136 (18.4%)

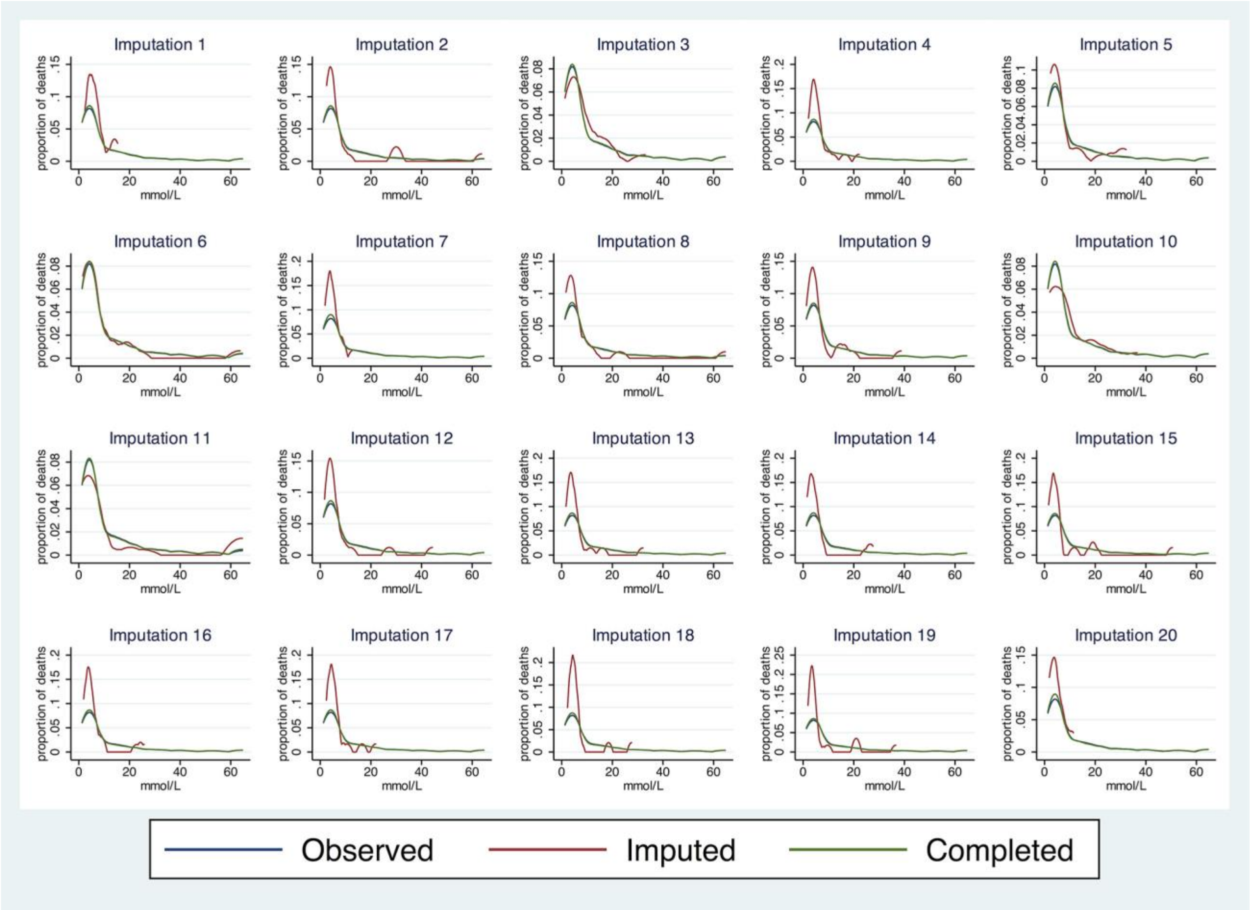

Technical Appendix Figure 7. Blood urea nitrogen (BUN): missing = 6.5% (20/309); Ebola+: 2/173 (1.2%); Ebola–: 18/136 (13.2%)

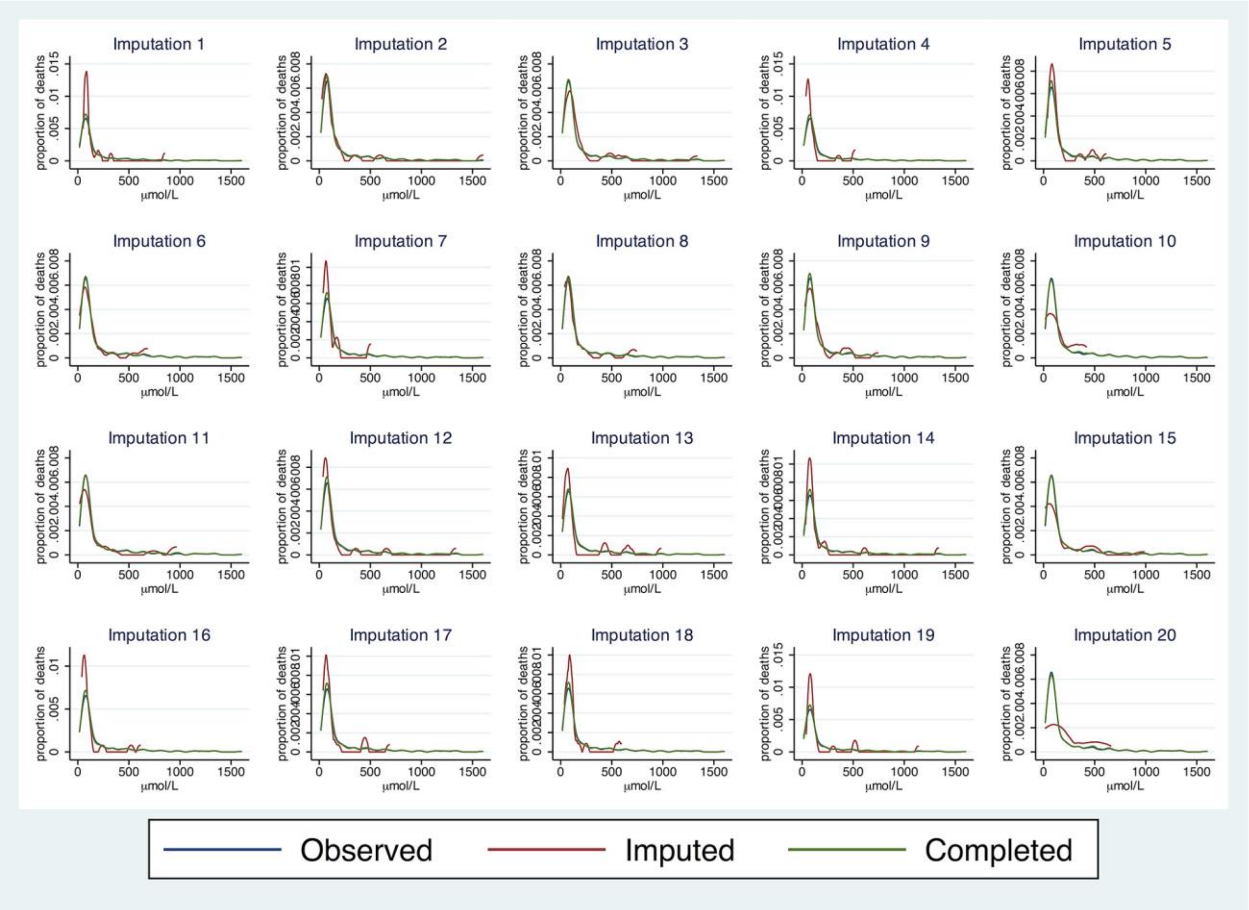

Technical Appendix Figure 8. Creatinine: missing = 7.8% (24/309); Ebola+: 3/173 (1.7%); Ebola–: 21/136 (15.4%)

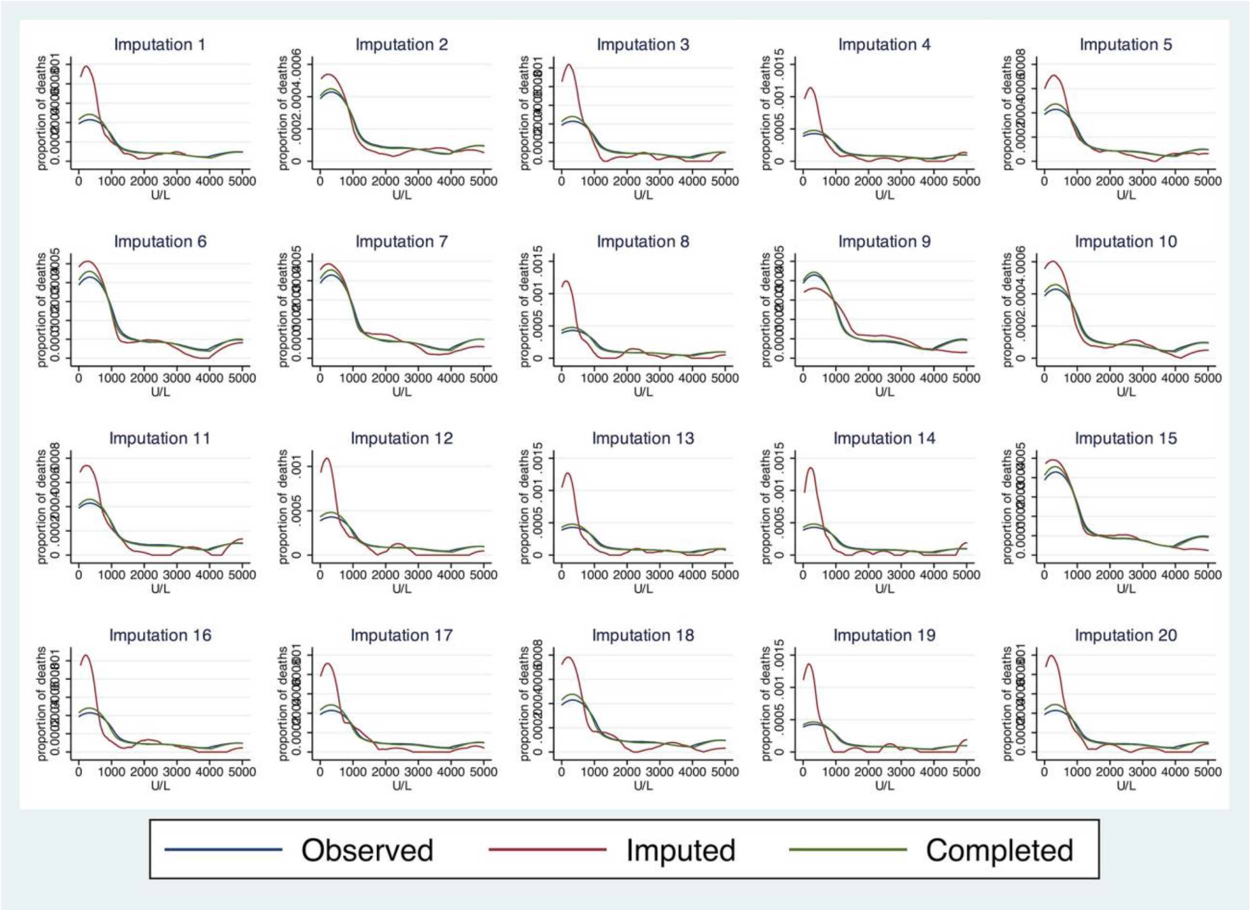

Technical Appendix Figure 9. Creatine kinase (CK): missing = 11.7% (36/309); Ebola+: 11/173 (6.4%); Ebola–: 25/136 (18.4%)

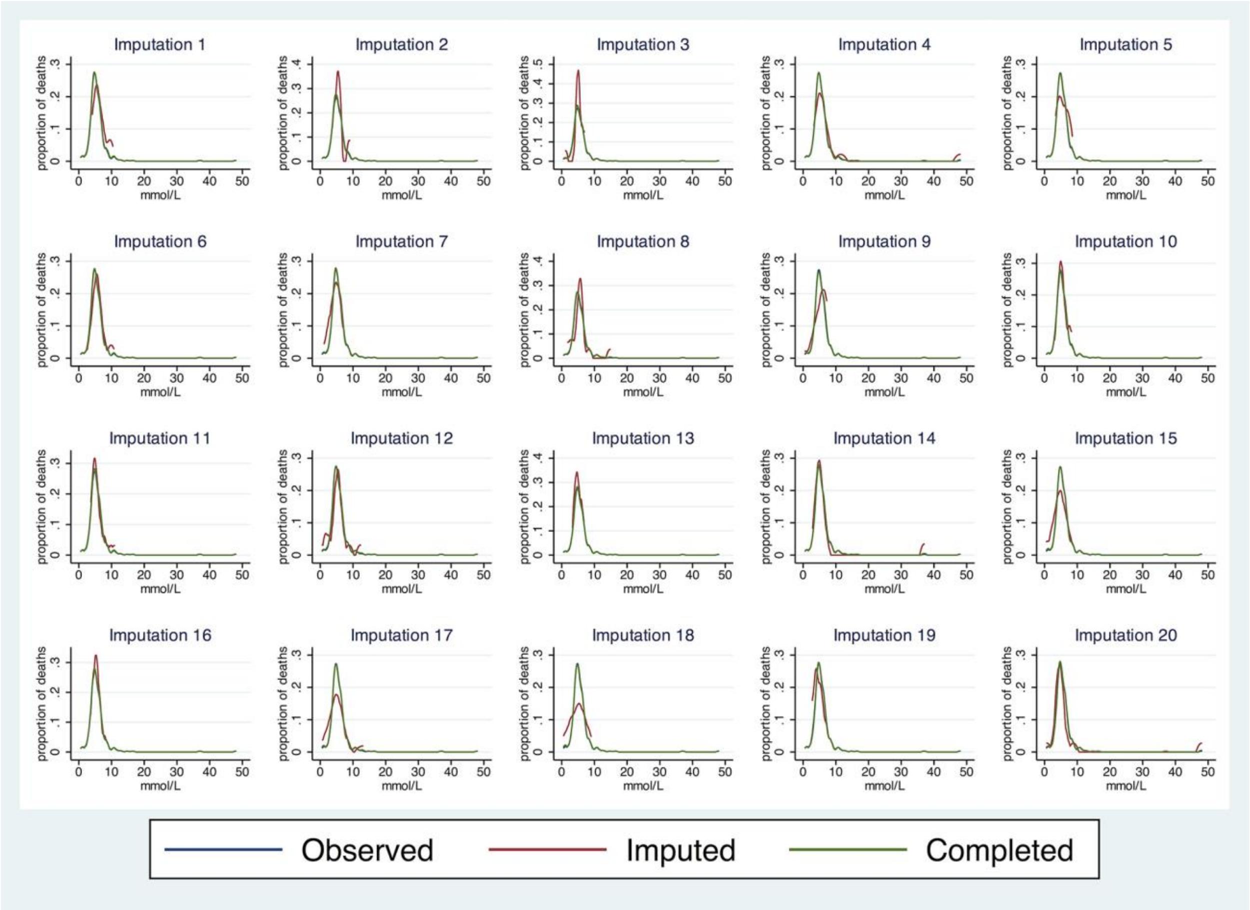

Technical Appendix Figure 10. Glucose: missing = 5.2% (16/309); Ebola+: 1/173 (0.6%); Ebola–: 15/136 (11.0%)

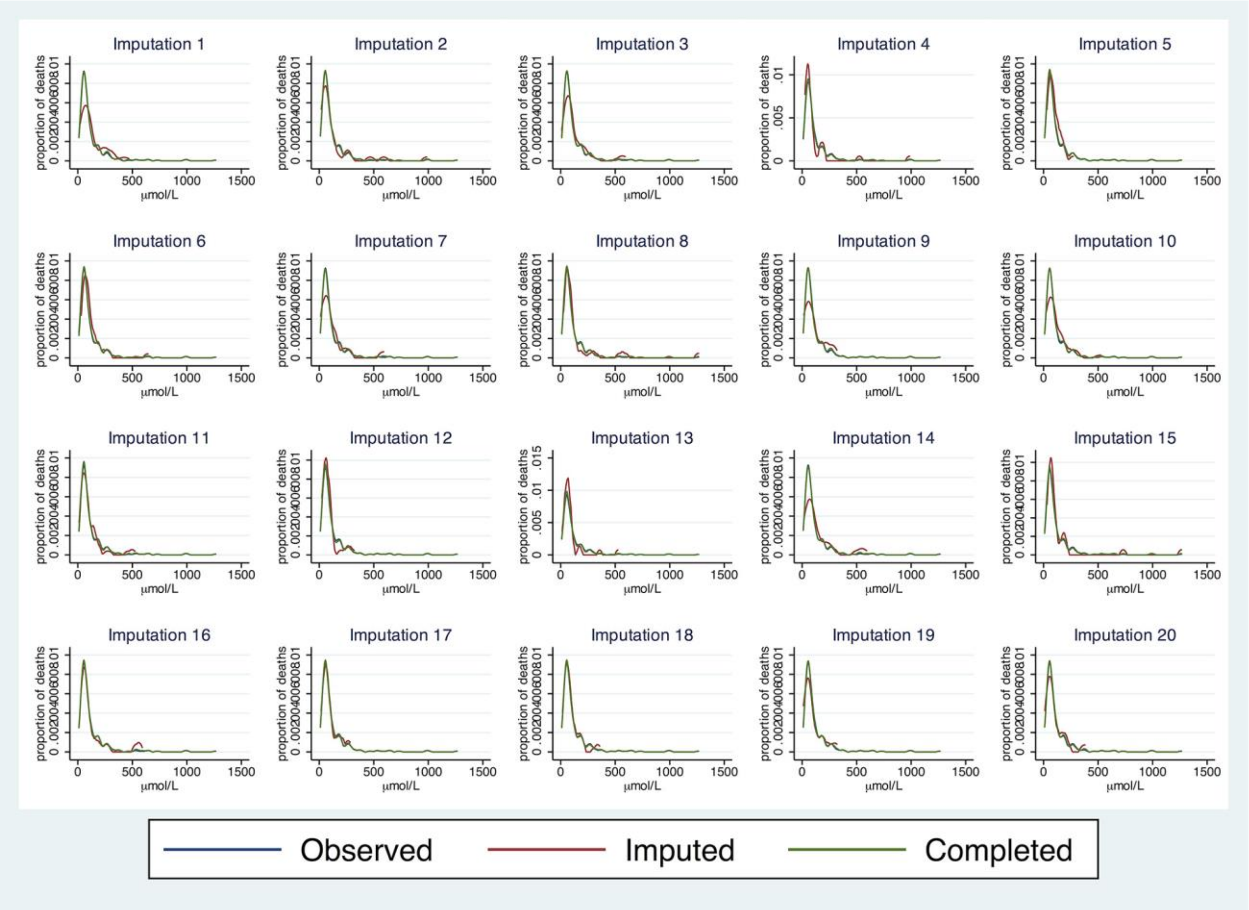

Technical Appendix Figure 11. Amylase: missing = 11.7% (36/309); Ebola+: 10/173 (5.8%); Ebola–: 26/136 (19.1%)

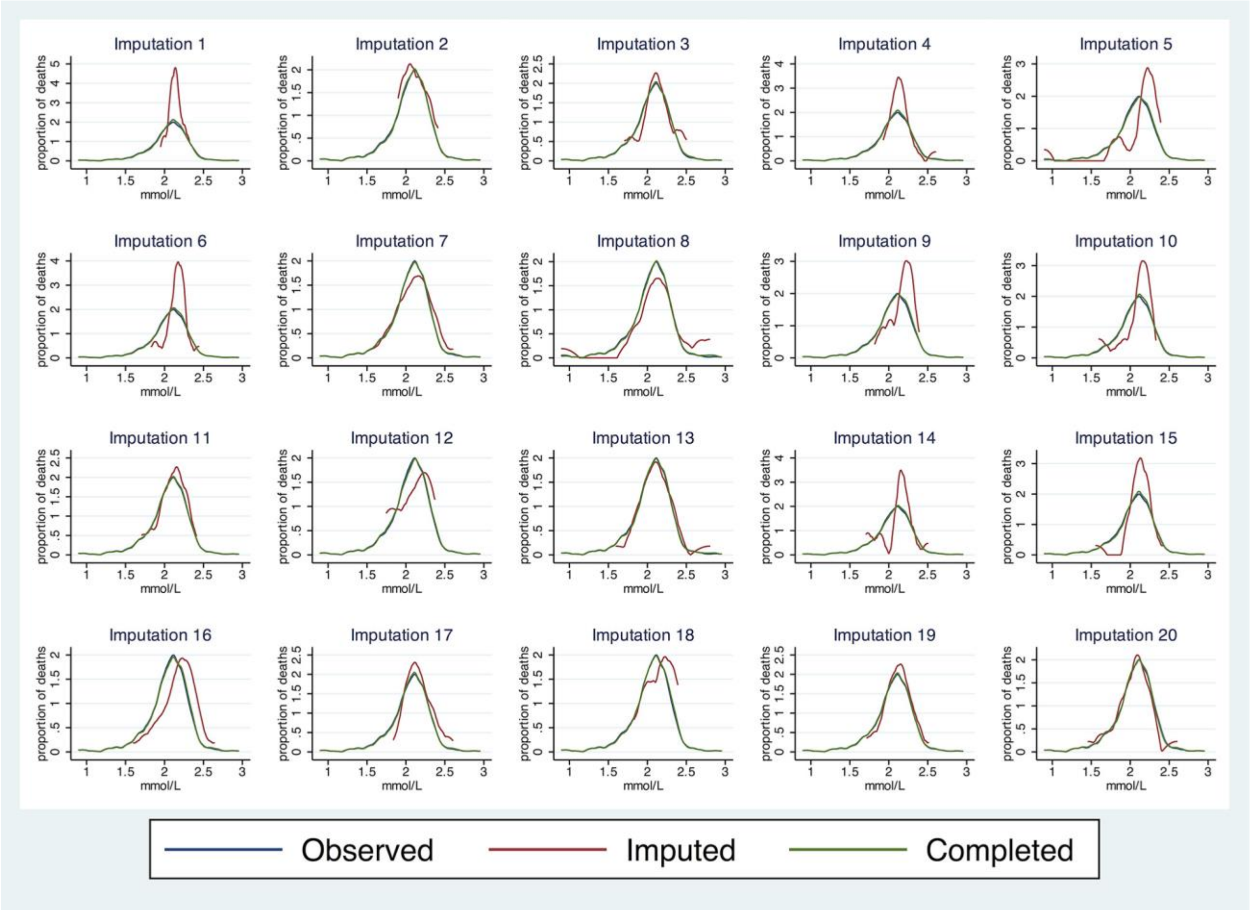

Technical Appendix Figure 12. Total calcium: missing = 11.7% (36/309); Ebola+: 1/173 (0.6%); Ebola–: 16/136 (11.8%)

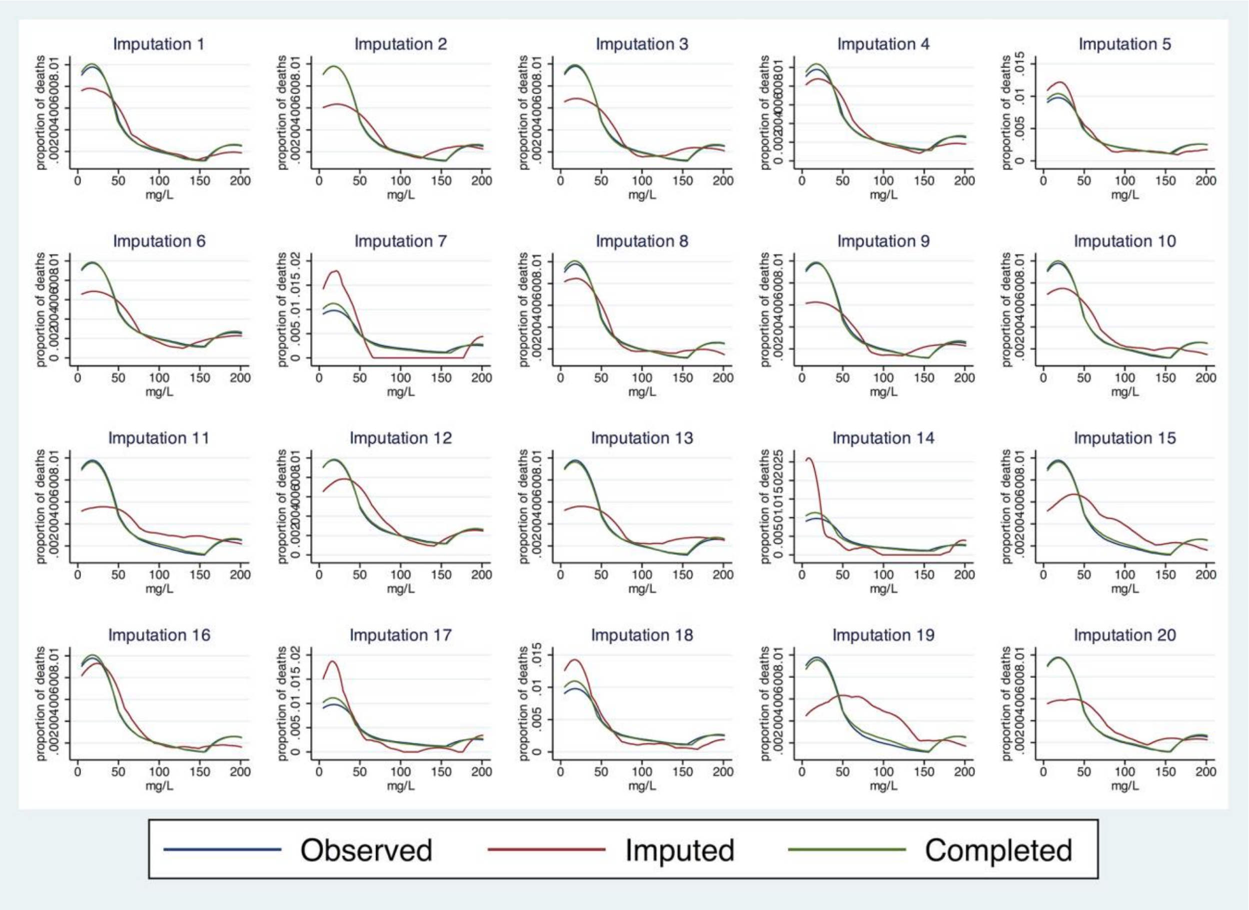

Technical Appendix Figure 13. C-reactive protein (CRP): missing = 11.7% (36/309); Ebola+: 10/173 (5.8%); Ebola–: 26/136 (19.1%)

Laboratory Test Regression Analyses

Technical Appendix Table 1. Patient laboratory test values by Ebola virus disease status*

	EVD negative (%)	EVD positive (%)	Univariable logistic regression†	
			Coefficient (95% CI)	p-value
Sodium <128 mmol/L	27/120 (22.5)	56/169 (33.1)	0.78 (0.11 to 1.45)	0.023
Potassium <3.6 mmol/L	16/115 (13.9)	30/143 (21.0)	0.47 (–0.27 to 1.22)	0.211
Alanine aminotransferase >48 U/L	19/109 (17.4)	137/151 (90.7)	3.22 (2.36 to 4.08)	<0.001
Aspartate aminotransferase >35 U/L	62/109 (56.9)	130/137 (94.9)	2.61 (1.60 to 3.61)	<0.001
Albumin <33 g/L	80/121 (66.1)	94/170 (55.3)	–0.19 (–0.75 to 0.38)	0.521
Total bilirubin >27 μmol/L	18/111 (16.2)	16/135 (11.9)	–0.81 (–1.68 to 0.06)	0.069
Blood urea nitrogen >7.9 mmol/L	29/118 (24.6)	76/170 (44.7)	0.80 (0.19 to 1.40)	0.010
Creatinine >106 μmol/L	38/115 (33.0)	80/165 (48.5)	0.39 (–0.18 to 0.96)	0.179
Creatine kinase >380 U/L	22/111 (19.8)	116/149 (77.9)	2.43 (1.76 to 3.10)	<0.001
Glucose >6.6 mmol/L	21/121 (17.4)	33/171 (19.3)	0.30 (–0.44 to 1.04)	0.425
Amylase >97 U/L	25/110 (22.7)	65/151 (43.1)	0.62 (0.00 to 1.24)	0.051
Total calcium <2 mmol/L	23/120 (19.2)	70/169 (41.4)	1.18 (0.52 to 1.85)	0.001
C-reactive protein >7.5 mg/L	73/110 (66.4)	116/150 (77.3)	0.62 (–0.01 to 1.25)	0.053

*CI, confidence interval; EVD, Ebola virus disease
†Based on imputed results for missing laboratory tests

Reclassification Table for Ebola Symptom-Based Risk (ESR) Score versus the Ebola Symptom- and Laboratory-Based Risk (ESLR) Score

Technical Appendix Table 2. High-, medium-, and low-risk reclassification table for ESR versus ESLR scores*

		Ebola symptom and laboratory-based risk (ESLR) score		
		# Low (%)	# Medium (%)	# High (%)
EVD-positive patients (n = 173)				
	# Low (%)	8 (4.6)	0 (0.0)	0 (0.0)
	# Medium (%)	2 (1.2)	3 (1.7)	1 (0.6)
	# High (%)	19 (11.0)	24 (13.9)	116 (67.1)
EVD-negative patients (n = 136)				
	# Low (%)	50 (36.8)	12 (8.8)	0 (0.0)
	# Medium (%)	7 (5.1)	23 (16.9)	4 (2.9)
	# High (%)	2 (1.5)	12 (8.8)	26 (19.1)

*EVD = Ebola virus disease
